# Coordinated transcriptional and post-transcriptional epigenetic regulation during skeletal muscle development and growth in pigs

**DOI:** 10.1186/s40104-022-00791-3

**Published:** 2022-12-01

**Authors:** Du Zhang, Shumei Wu, Xinxin Zhang, Shuqiang Ren, Zhonglin Tang, Fei Gao

**Affiliations:** 1 Genome Analysis Laboratory of the Ministry of Agriculture, Agricultural Genomics Institute at Shenzhen, Chinese Academy of Agricultural Sciences, Shenzhen, 518000 China; 2 College of Animal Sciences, South China Agricultural University, Guangdong Provincial Key Lab of Agro-animal Genomics and Molecular Breeding, National Engineering Research Center for Breeding Swine Industry, Guangzhou, 510642 China; 3 Kunpeng Institute of Modern Agriculture at Foshan, Chinese Academy of Agricultural Sciences, Foshan, 528226 China; 4 GuangXi Engineering Centre for Resource Development of Bama Xiang Pig, Bama, 547500 China; 5 Comparative Pediatrics and Nutrition, Department of Veterinary and Animal Sciences, Faculty of Health and Medical Sciences, University of Copenhagen, Copenhagen, Denmark

**Keywords:** DNA methylation, Epigenetic modification, Epigenomic analysis, 5mC regulators, m^6^A methylation, m^6^A regulators, Myogenesis

## Abstract

**Background:**

N6-methyladenosine (m^6^A) and DNA 5-methylcytosine (5mC) methylation plays crucial roles in diverse biological processes, including skeletal muscle development and growth. Recent studies unveiled a potential link between these two systems, implicating the potential mechanism of coordinated transcriptional and post-transcriptional regulation in porcine prenatal myogenesis and postnatal skeletal muscle growth.

**Methods:**

Immunofluorescence and co-IP assays were carried out between the 5mC writers and m^6^A writers to investigate the molecular basis underneath. Large-scale in-house transcriptomic data were compiled for applying weighted correlation network analysis (WGCNA) to identify the co-expression patterns of m^6^A and 5mC regulators and their potential role in pig myogenesis. Whole-genome bisulfite sequencing (WGBS) and methylated RNA immunoprecipitation sequencing (MeRIP-seq) were performed on the skeletal muscle samples from Landrace pigs at four postnatal growth stages (days 30, 60, 120 and 180).

**Results:**

Significantly correlated expression between 5mC writers and m^6^A writers and co-occurrence of 5mC and m^6^A modification were revealed from public datasets of C2C12 myoblasts. The protein-protein interactions between the DNA methylase and the m^6^A methylase were observed in mouse myoblast cells. Further, by analyzing transcriptome data comprising 81 pig skeletal muscle samples across 27 developmental stages, we identified a 5mC/m^6^A epigenetic module eigengene and decoded its potential functions in pre- or post-transcriptional regulation in postnatal skeletal muscle development and growth of pigs. Following integrative multi-omics analyses on the WGBS methylome data and MeRIP-seq data for both m^6^A and gene expression profiles revealed a genome/transcriptome-wide correlated dynamics and co-occurrence of 5mC and m^6^A modifications as a consequence of 5mC/m^6^A crosstalk in the postnatal myogenesis progress of pigs. Last, we identified a group of myogenesis-related genes collaboratively regulated by both 5mC and m^6^A modifications in postnatal skeletal muscle growth in pigs.

**Conclusions:**

Our study discloses a potential epigenetic mechanism in skeletal muscle development and provides a novel direction for animal breeding and drug development of related human muscle-related diseases.

**Supplementary Information:**

The online version contains supplementary material available at 10.1186/s40104-022-00791-3.

## Introduction

The skeletal muscle is the largest organ of mammals and is crucial to the metabolism and energy balance of the whole body [1]. Disruption of muscle fiber development may lead to human muscular diseases, e.g., muscle atrophy [2, 3] and a decrease in livestock meat quality and meat production [4]. Pigs represent a major source of meat production worldwide and an ideal animal model in myogenesis studies [5, 6]. Deciphering the molecular mechanisms that regulate skeletal muscle development and growth is significant for both animal husbandry and biomedicine.

The spatiotemporal expression of gene functions in skeletal muscle development and growth is tightly regulated by dynamic epigenetic modifications [7, 8]. DNA 5-methylcytosine (5mC) and RNA N6-methyladenosine (m^6^A) are two typical epigenetic modifications that play essential roles in transcription and post-transcription regulation, respectively [9–11]. Recently, many studies have explored the epigenetic mechanisms underlying skeletal muscle growth and development in mammals, including chromatin landscape [12, 13], microRNA [14, 15], circRNA [16, 17], DNA and histone modifications [14, 18]. The discovery of the RNA methylation system in post-transcriptional regulation of myoblast differentiation and proliferation provides another layer of information for understanding skeletal muscle development and growth [19, 20]. Interestingly, one previous study reported significant positive correlations in genetic variation and gene expression of 5mC and m^6^A regulators, which include a group of proteins responsible for 5mC and m^6^A modification addition (writers), identification (readers) and removal (erasers) from DNA or RNA sequence, based on analyzing the gene expression profiles of 11,080 samples from 33 kinds of human cancers in the TCGA database [21]. Another study also indicated for quantitative correlation between 5mC and m^6^A modifications in the genome of tomatoes [22]. These studies implicated the potential mechanism of coordinated transcriptional and post-transcriptional regulation in various biological processes, which provides new possibilities for deciphering the mechanism of skeletal muscle development and growth.

Previously, we have characterized DNA methylome dynamics of 27 developmental stages in pig skeletal muscle development and revealed the involvement of the DNA methylation/SP1/IGF2BP3 axis in skeletal myogenesis [23]. In addition, we also profiled the RNA methylation landscape during prenatal skeletal muscle development, using the same collection of samples [20]. In the present study, we hypothesized that the transcriptional (5mC) and post-transcriptional (m^6^A) regulation of hub genes of porcine skeletal muscle development and growth are tightly coordinated via protein-protein interaction of 5mC and m^6^A methylases. To test this, we first demonstrated the genome/transcriptome-wide association between 5mC and m^6^A modification as well as their regulators’ expression, following with co-IP and immunofluorescence experiments to confirm the interaction between 5mC and m^6^A methylases, in the C2C12 myoblasts. Next, we evaluated the key 5mC/m^6^A regulators that played an important role in regulating prenatal and postnatal skeletal muscle development and identified a group of essential target genes whose transcription and translation are coordinately regulated by these regulators. Our study uncovered a novel epigenetic mechanism in skeletal muscle development and provided novel directions for investigating animal breeding, muscle biology, and related human diseases.

## Materials and methods

### Cell culture

For immunofluorescence experiments, C2C12 cells were cultured in DMEM supplemented with 10% FBS (Gibco, California, USA) in a humidified incubator with 5% CO_2_ at 37 °C. For co-IP assays, the cells were transferred to DMEM containing 2% horse serum (Gibco, California, USA) and induced to differentiate for 4 d.

### Immunofluorescence

C2C12 cells were fixed with 4% paraformaldehyde for 15 min at room temperature. After permeabilization with 0.25% Triton X-100 for 10 min, the cells were blocked by using 1% bovine serum albumin for 30 min. Then, the cells were incubated overnight with Anti-METTL3 antibody (67733–1-Ig; Protentech, Wuhan, China), and Anti-DNMT3A antibody (ab228691; Abcam, Cambridge, England),or Anti-DNMT3B antibody (ab227883; Abcam, Cambridge, England) diluted in PBST containing 1% bovine serum albumin at 4 °C. The cells were incubated with Alexa Fluor 647 donkey anti-mouse IgG (H + L), (A-31571; Invitrogen, MA, USA) or Alexa Fluor 488 donkey anti-rabbit IgG (H + L) (A-21206; Invitrogen, MA, USA) for 1 h at room temperature. Nuclei were stained with DAPI for 1 min. The cells observed microscopically using an A1HD25 confocal microscope (Nikon Instruments, Tokyo, Japan).

### Co-IP assays

The differentiated C2C12 cells lyzed according to the instructions of the Co-Immunoprecipitation Kit (BersinBio, Guangzhou, China). Anti-Mettl3 antibody (ab195352), Anti-METTL14 antibody (ab252562), Anti-WTAP antibody (ab195380), Anti-DNMT1 antibody (ab19905), Anti-DNMT3A antibody (ab228691) and Anti-DNMT3B antibody (ab227883) were used for IP (Abcam, Cambridge, England). Nonspecific IgG antibodies (ab24584; Abcam, Cambridge, England) precipitated the complex as a control group. Total proteins from C2C12 cells were extracted and incubated overnight with IgG or the Co-IP antibodies (5 μg) at 4 °C, while the extracted proteins without any antibodies were used as Input control. Next, 30 μL of Protein A/G PLUS-Agarose (sc-2027; Santa Cruz Biotechnology, California, USA) was added and incubated for 2 h at 4 °C to form an immune complex. Following centrifugation for 4 min at 3000 r/min at 4 °C, the Protein A/G Plus-Agarose beads were washed 4 times with 1 mL of lysate and boiled in the appropriate protein loading buffer for 5 min. Centrifugation at 3000 r/min again, the supernatants were collected to a new tube for Western blot analysis.

### Western blot

The total proteins from C2C12 cells were lysed in RIPA lysis buffer supplemented with a protease inhibitor cocktail (4693124001; Roche, Mannheim, Germany). The membranes were blocked with 5% BSA for 1 h at room temperature and subsequently probed with primary antibodies overnight at 4 °C. the following dilutions were used for each antibody: METTL3, METTL14, WTAP, DNMT1, DNMT3A and DNMT3B (all 1:1000; Abcam, Cambridge, England). The membranes were then washed with PBS-Tween and incubated for 50 min with horseradish peroxidase-conjugated secondary antibodies (SA00001–1; Proteintech, Wuhan, China). Protein bands were detected after treatment with the SuperSignal west Femto agent (34094; Thermo Scientific, Waltham, USA).

### Tissue sample collection

The skeletal muscle (*longissimus dorsi*, LD) samples were collected from Landrace pigs at four developmental stages, including postnatal days 30, 60, 120, and 180 (abbreviated as LD30, LD60, LD120, and LD180), as we previously reported [23]. The experimental pigs were allowed access to feed and water ad libitum and were housed under identical conditions before slaughtering. After copulation with the boar, the sows and piglets were sacrificed at a commercial slaughter house at the selected stages. At each stage, skeletal muscles from two unrelated pigs were harvested as biological replicates. All samples were stored immediately in liquid nitrogen until further use. All animal procedures were performed according to protocols of the Chinese Academy of Agricultural Sciences and the Institutional Animal Care and Use Committee.

### MeRIP sequencing

Total RNA was immediately isolated using Trizol reagent according to the manufacturer’s instructions (AM8740; Invitrogen, Carlsbad, USA). All samples were treated with DNase (M0303L; NEB, Ipswich, USA) to avoid DNA contamination. Profiling of m^6^A sites for each mRNA was performed as described in a previous study [24]. For isolation of mRNA, total RNA was subjected to two rounds of purification using oligo (dT)-coupled magnetic beads according to the manufacturer’s instructions (Invitrogen, Carlsbad, USA). mRNA samples were fragmented into ~ 100-nucleotide fragments by incubating in fragmentation buffer (Invitrogen, Carlsbad, USA) at 90 °C for 1 min. The fragmented mRNA (approximately 50 ng) was used to construct the input library. The remaining mRNA fragments were incubated for 4 h at 4 °C with 3 mg of anti-m^6^A polyclonal antibody (202003; Synaptic Systems, Goettingen, Germany) combined with protein A beads (Invitrogen, Carlsbad, USA) at room temperature for 1 h in immunoprecipitation purification (IPP) buffer (150 mmol/L NaCl, 0.1% NP-40, 10 mmol/L Tris-HCl). Bound mRNA was eluted from the beads with 0.5 mg/mL of m^6^A in IPP buffer. Eluted mRNA was precipitated using an ethanol-sodium acetate solution and glycogen (R0551; Thermo Scientific, Waltham, USA) overnight at − 80 °C. mRNA was resuspended in water and used for library generation. Input mRNA and IP mRNA sequencing libraries were generated using a Vazyme mRNA-seq Kit for Illumina (NR601; Vazyme, Nanjing, China). Sequencing was performed using an Illumina HiSeq X Ten system (Illumina, San Diego, USA)**.**

### MeRIP sequencing data analysis

For each sample, paired-end reads were used for bioinformatics analysis. Raw data quality control was performed using FastQC software (version 0.11.3). Sequencing data were trimmed using Trimmomatic (version 0.36) [25] with default parameters to remove the adapter and low-quality data. These high-quality reads were mapped against the ensemble pig genome (Sscrofa11.1) using hisat2 software (version 2.0.1) [26]. m^6^A-modified RNA regions (m^6^A peaks) and differentially m^6^A methylated peaks were identified using exomePeak software (version 2.16.0) [27, 28]. m^6^A peaks identified in two biological replicates were merged and regarded as highly enriched and selected for further analysis (*P*-value < 0.05).

### Identification of hub 5mC/m^6^A regulators based on the topology of the co-expression networks

Methods for hub 5mC/m^6^A regulators and epigenetic module eigengenes (EME) identification were described in Chen et al.’s paper [21]. To identify hub 5mC and m^6^A regulators for pig skeletal muscle, we introduced the concept of “module” from the weighted gene co-expression network analysis (WGCNA) algorithm and treated the 41 5mC and m^6^A regulators as a module [29]. The overall expression level of the module was summarized as the module eigengene by the “moduleEigengenes” function in the R package WGCNA. We further calculated the module membership (i.e., module eigengene-based intramodular connectivity) as the correlation between the expression value of a given 5mC/m^6^A regulator and the module eigengene. Hub 5mC/m^6^A regulators were then defined as those that achieved a module membership greater than 0.7. The summary expression level of the identified hub 5mC/m^6^A regulators was again calculated as EME for pig skeletal muscle.

### EME correlated co-expression analysis

Genes with the top 25% variant RNA expression level among all the 81 pig skeletal muscle samples were selected for co-expression analysis. Then we identified the co-expression modules by the WGCNA algorithm with default parameters. Correlation analysis was conducted between the EME vector and the identified co-expression modules. Modules with *r* > 0.8 and *P*-value < 0.05 were regarded as EME correlated co-expression modules for further analysis.

### 5mC/m^6^A interaction effects analysis

To find out 5mC/m^6^A interaction effects in the 80 up-regulated genes in the postnatal stages, we recruited the general linear model (GLM) using the postnatal days, 5mC methylation level, enrich score of m^6^A peaks and 5mC/m^6^A interaction item as explanatory variables:

$${Log}_2\left( FPKM+1\right)=S+X+Y+X\times Y+\varepsilon$$

Here, *S* denotes the postnatal days, *X* is the enrich score of an m^**6**^A peak, *Y* is the 5mC methylation level of the m^6^A peak, *X × Y* is the interaction item, and *ε* is the random variable. Additionally, the enrich score of a specified m^6^A peak is calculated as:

$$Escore=\frac{RPM_{IP}}{RPM_{Input}}$$

Where:

$$RPM=\frac{A\times {10}^6}{\sum A}$$

Here, IP is the MeRIP sample and Input is the corresponding control, *A* is the read counts mapped to a specified peak region in a sample, and ∑*A* is the total mapped read counts in a sample. Finally, regions or genes with a *P*-value < 0.05 for the interaction item were selected as of 5mC/m^6^A modification interaction effect regions or genes. All the fitted GLM were constructed by the lm function in R.

### Protein-protein interaction analysis

In addition, we identified the protein-protein interactions among the co-expression genes in the EME negative correlated module based on the STRING database [30].

### Function enrichment analysis

Gene list enrichment analysis was performed using the online tool Enrichr [31]. Enrichment results from MSigDB hallmark pathway, GO biological process, MGI mammalian phenotype level 4, and ARCHS4 TFs co-expression with a *P*-value < 0.05 were considered nominally significant.

### Public data access and analysis

The RNA-seq data of C2C12 myoblasts were downloaded from GEO datasets (GSE131842). Expression levels of DNA 5mC writers (DNMT3A, DNMT3B, and DNMT1) and RNA m^6^A writers (METTL3, METTL14, and WTAP) were extracted and followed with Pearson’s correlation analysis. The RRBS and MeRIP-seq data of C2C12 cells were also downloaded from GEO datasets with accession numbers GSE131842 and GSE154720, respectively. The CpG methylation matrix and the m^6^A peak tables were directly used in the further co-enrichment analysis. For 5mC/m^6^A co-occurrence analysis, we split the upstream 5 kb, the m^6^A peak region, and the downstream 5 kb sequence into 20, 10, and 20 bins with the same length, respectively. Then the bins covered with no less than 20 CpG sites were selected for average methylation level calculation and visualization. In total, 81 RNA-seq data from 27 prenatal and postnatal developmental stages (including embryonic days 33, 40, 45, 50, 55, 60, 65, 70, 75, 80, 85, 90, 95, 100, 105 and postnatal days 0, 9, 20, 30, 40, 60, 80, 100, 120, 140, 160 and 180, each stage with three replicates) and 8 WGBS data from the four postnatal developmental stages (postnatal days 30, 60, 120 and 180, each stage with two replicates) were obtained from a previously in-house study on pig skeletal muscle development [23]. The whole design of this study was clarified in Additional file 1: Fig. S1.

## Results

### Protein-protein interactions between 5mC and m^6^A writers

**Fig. 1:**
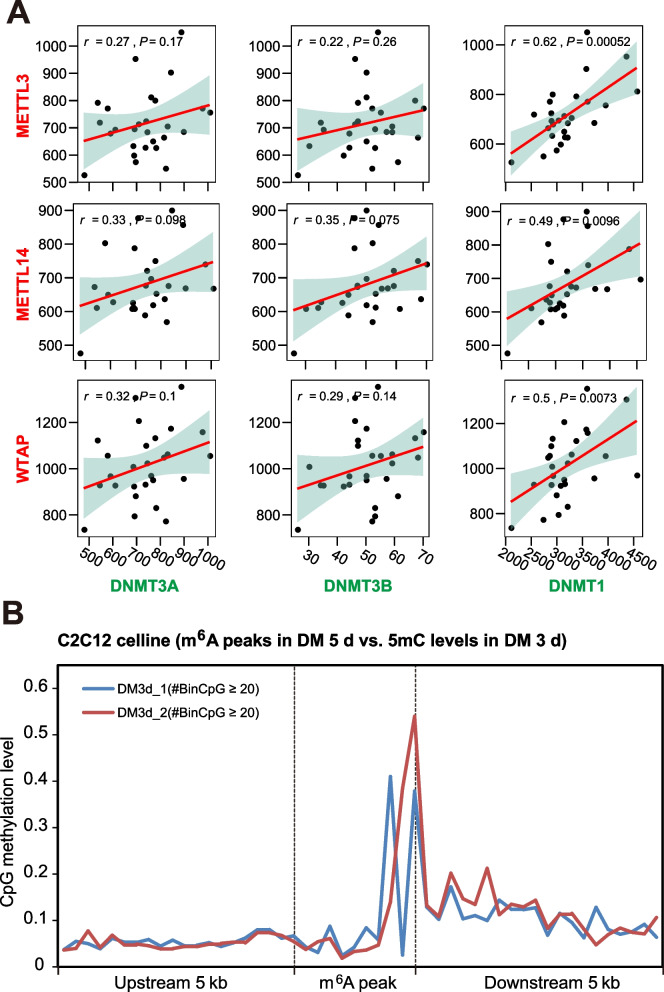
Association between DNA 5mC and RNA m^6^A regulators in skeletal muscle. **A** Correlation between the mRNA expression levels of 5mC writers (DNMT3A, DNMT3B, and DNMT1) and m^6^A writers (METTL3, METTL14, and WTAP) in C2C12 cell lines (27 samples from GSE131842). **B** Overlapping enrichment of CpG methylation in the m^6^A peaks in the C2C12 cell line (m^6^A data from GSE154720 and CpG methylation data from GSE131842). The m^6^A peaks, upstream 5 kb regions, downstream 5 kb regions were divided into 10, 20, 20 bins, respectively. Bins covered with no less than 20 CpG sites were selected for average methylation level calculation and visualization

To investigate the coordinated relations between 5mC and m^6^A writers, we checked the patterns of expression correlation between DNA 5mC writers (DNMT3A, DNMT3B, and DNMT1) and RNA m^6^A writers (METTL3, METTL14, and WTAP) in mouse myoblast cells (C2C12) after differentiation for 0, 12, 24, 48 and 60 h (3 biological replicates for each time point). Positive correlations were unveiled between the mRNA level of 5mC and m^6^A writers (Fig. 1A). In addition, we collected genome-wide 5mC and m^6^A data of a C2C12 muscle cell line (based on RRBS and MeRIP-seq data from myoblasts that had been in differentiation media for 5 d and 3 d, respectively) and found a significantly higher methylation level in the m^6^A peaks than in the up- or down-stream regions (Fig. 1B), which indicated co-occurrence of 5mC modification in the genome and m^6^A modification in the corresponding transcriptome.

**Fig. 2:**
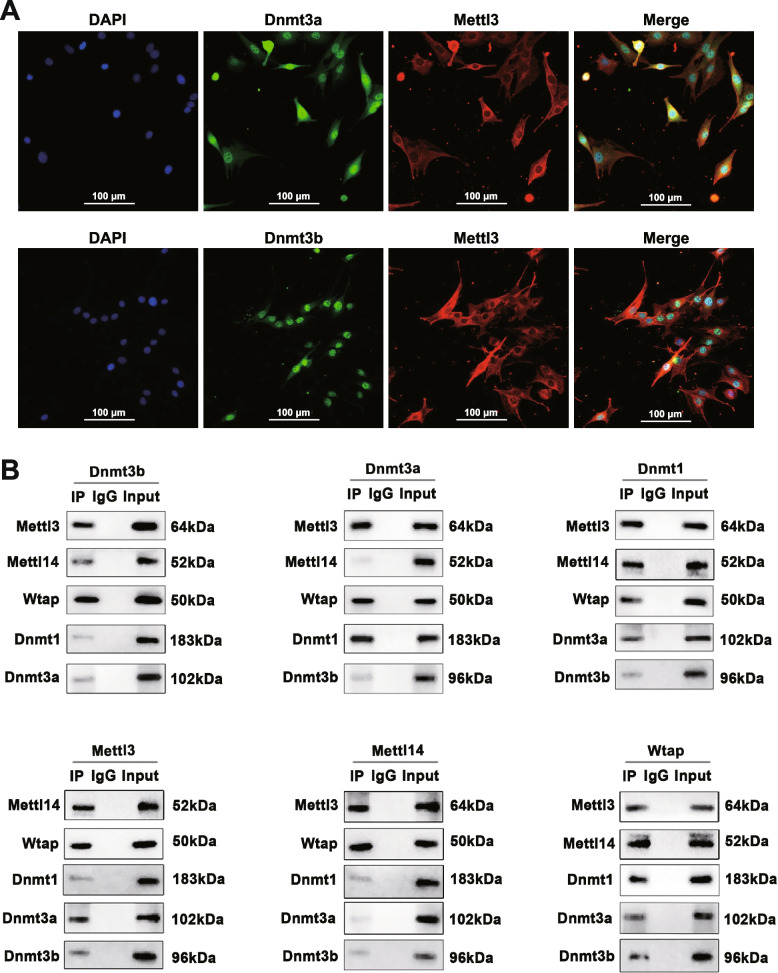
Interactions between the RNA methylase and the DNA methylase. **A** Co-IP products were validated by Western blotting with DNMT1/DNMT3A/ DNMT3B (top) or with METTL3/METTL14/WTAP (bottom) as bait. **B** Co-localization of METTL3 with DNMT3A (top) or DNMT3B (bottom) in the nuclei of C2C12 cells

We hypothesize that there are protein-protein interactions among the writers of these two types of epigenetic modifications in skeletal muscle. We performed immunofluorescence assays and showed that METTL3 co-localizes with DNMT3A and DNMT3B in the nucleus of C2C12 cells (Fig. 2A), though most of the METTL3 localizes in the cytoplasm. Further, we performed co-immunoprecipitation (co-IP) assays on the C2C12 cells differentiated for 4 d using the main DNA methylases (DNMT1/DNMT3A/DNMT3B) as bait proteins (see Materials and methods). We purified the preys and confirmed the known m^6^A methylases of (METTL3/METTL14/WTAP) by Western blot analyses (Fig. 2B). To minimize the biases from the experiments, we reversed the co-IP experiments by using RNA methylases as bait proteins and testing the DNA methylases via Western blot. Thereby, the relative intensity of the interaction was calculated as the average values of the two experiments (Additional file 1: Fig. S2). Previous studies have demonstrated the close interactions between the three 5mC methylases [32, 33] and between the three m^6^A methylases [34], respectively. We confirmed such close interactions in the present study and further revealed significant interactions between the 5mC and m^6^A methylases. Specifically, the relative intensities of the interactions between 5mC and m^6^A methylases are comparable to the interactions among the three individual m^6^A methylases.

### Significantly correlated 5mC and m^6^A regulators in myogenesis regulation during prenatal and postnatal muscle development of pigs

**Fig. 3:**
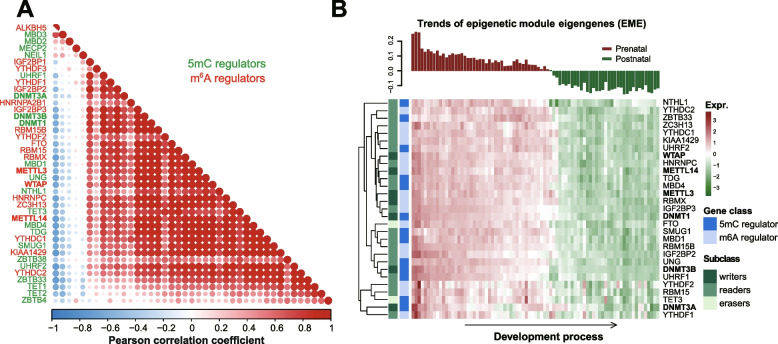
Indentification of 5mC/m^6^A epigenetic module eigengenes (EME) in skeletal muscle development of pigs. **A** Correlation between the mRNA expression levels of 5mC regulators and m^6^A regulators in pig skeletal muscle (In house data). The Pearson correlation coefficients are shown. **B** Expression pattern of the identified EME (top) and the expression heatmap of the 29 hub 5mC/m^6^A regulators (bottom)

To investigate the potential 5mC/m^6^A crosstalk in the skeletal muscle, we first obtained gene expression datasets from a previously published in-house study containing porcine skeletal muscle samples from 27 developmental stages [23]. We checked the patterns of expression correlation between 21 5mC regulators and 20 m^6^A regulators, including epigenetic writers, readers and erasers that were curated in a previous study [21]. We observed highly correlated expression patterns for the same class of regulators and even higher correlations between the expression of 5mC and m^6^A regulators in pig skeletal muscle (Fig. 3A), which is in accordance with previous findings in human cancer [21].

**Fig. 4:**
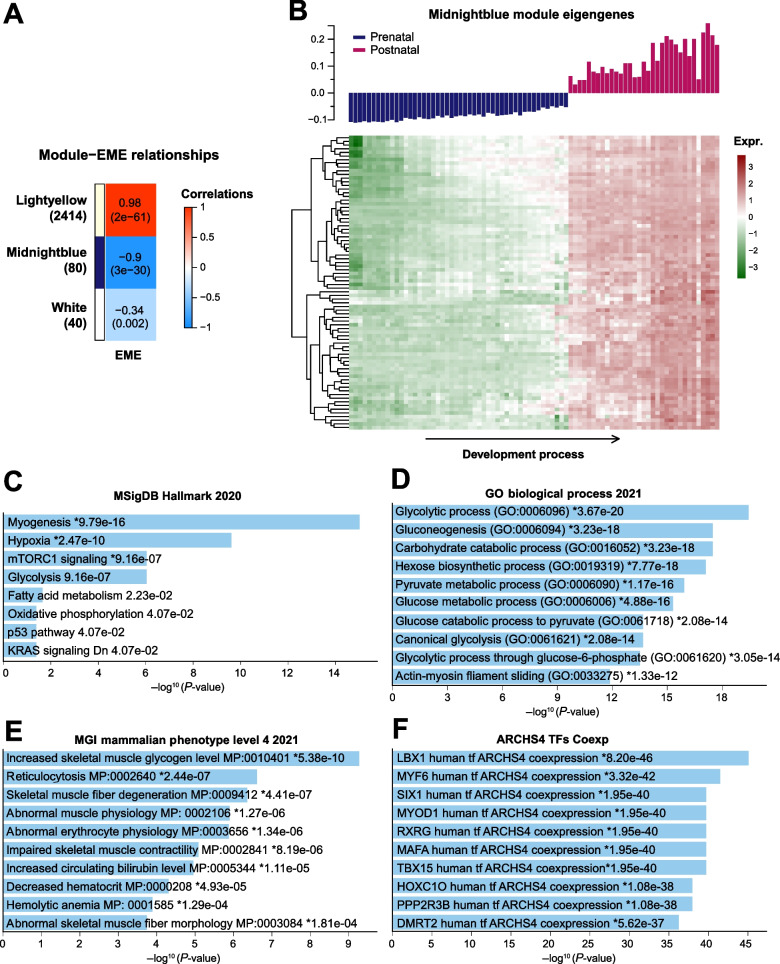
Potential regulation role of m^6^A/5mC EME in postnatal skeletal muscle development of pigs. **A** Correlation analysis between the EME and the co-expression modules identified in the top 25% variant expressing genes in pig skeletal muscle. Pearson correlation coefficient and the corresponding *P*-value were showed in the grid. Gene number in different modules was listed in the brackets underneath the corresponding module name. **B** Expression pattern of the EME negative correlated Midnightblue module (top) and the expression heatmap of each genes (bottom). **C**-**F** Enrichment analysis of Midnightblue module genes based on four different gene function databases

We further analyzed the co-expression gene modules from transcriptome data comprising 81 pig skeletal muscle samples across 27 developmental stages by applying a weighted gene co-expression network analysis (WGCNA) (Additional file 1: Fig. S3). Based on the co-expression gene network, 13 5mC regulators and 16 m^6^A regulators were identified as hub genes among the 41 m^6^A and 5mC regulators (see Materials and methods). These hub genes displayed dynamic transcriptional changes across the 27 developmental stages, i.e., mostly down-regulated in postnatal stages compared to prenatal stages (Fig. 3B). We then combined these hub genes to construct an epigenetic module eigengene (EME) to further explore the potential target genes under coordinated pre- and post-transcriptional regulation. We found three co-expression modules displayed a significant correlation with EME, among which the Lightyellow module shows a significantly positive correlation (Fig. 4A, *r* = 0.98, *P*-value = 3e-61, consisting of 2414 genes), while the Midnightblue module shows a significantly negative correlation (Fig. 4A, *r* = − 0.9, *P*-value = 3e-30, comprised of 80 genes). These results reveal that a group of genes might be under coordinated pre and post-transcriptional regulation by 5mC/m^6^A crosstalk. The expression heatmap indicated that these genes are highly divergent between prenatal and postnatal development and may be essential to embryonic or postnatal skeletal muscle development (Additional file 1: Fig. S4A and Fig. 4B).

Next, we examined the functional relevance of the EME correlated genes to myogenesis. Enrichment analysis reveals that the Ligthyellow module genes are involved in general RNA processes like splicing, translation and metabolism (Additional file 1: Fig. S4B). Interestingly, the Midnightblue module genes are involved in MSigDB hallmark pathways like myogenesis, hypoxia and glycolysis (Fig. 4C). GO analysis showed they participate in the biological process like glycolytic process, gluconeogenesis and carbohydrate catabolic process (Fig. 4D). They are also relevant to MGI mammalian phenotypes like increased skeletal muscle glycogen level, reticulocytosis, and skeletal muscle fiber degeneration (Fig. 4E). Co-expression analysis of transcript factors in the Midnightblue module revealed crucial known TF genes involved in human muscle development, including *LBX1*, *MYF6*, *SIX1*, and *MYOD1* (Fig. 4F). Moreover, we found genes in the Midnightblue module frequently interacted in the protein-protein interaction networks (Additional file 1: Fig. S5).

### DNA methylation and RNA m^6^A dynamics during the postnatal skeletal muscle development and growth in pig

To further address the 5mC and m^6^A dynamics across the muscle development and growth after birth, we obtained genome-wide 5mC and transcriptome-wide m^6^A data for the same set of skeletal muscle tissues from Landrace pigs at four postnatal time points (including postnatal days 30, 60, 120, and 180). The DNA methylome data was generated in the previous study [23]. In the present study, MeRIP-seq was conducted, obtaining an average of 36.89 million paired-end reads (150 bp × 2) for each sample, reaching an average mapping rate of 83.92% (range 73.47 to 91.28%) (Additional file 2: Table S1).

**Fig. 5:**
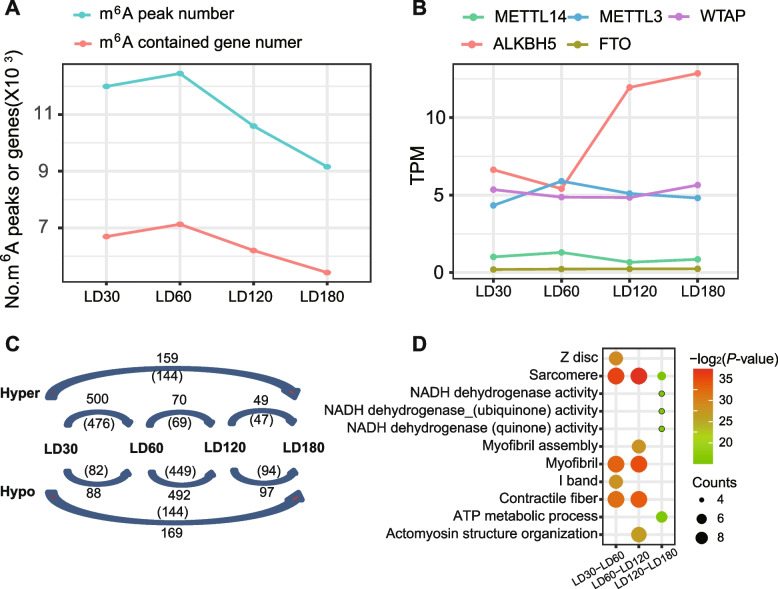
Dynamics of m^6^A epitranscriptome during postnatal skeletal muscle development. **A** The number of m^6^A peaks and genes containing m^6^A peaks in the four developmental stages. **B** mRNA expression profiles of m^6^A writers (METTL3/METTL14/WTAP) and erasers (ALKBH5/FTO). **C** Numbers of m^6^A hypermethylated or hypomethylated peaks (outside the bracket) and genes (inside the bracket) from inter-stage comparisons. **D** Top 5 GO terms enriched by the inter-stage differentially m^6^A modification genes

Across the four growth stages, m^6^A modifications were enriched in CDS and 3’UTR regions, especially in the TSS site (Additional file 1: Fig. S6). We then called the m^6^A consensus peaks and the m^6^A contained genes in the four growth stages. Intriguingly, both the m^6^A peak number (8969–12, 221 peaks) and the m^6^A modified gene number (5324–7003 genes) showed a slight increase in postnatal 60 d and then a persistently decrease onto the postnatal 180 d (Fig. 5A). Corresponding longitudinal gene expression analysis to the prominent m^6^A writers (METTL3, METTL4 and WTAP) and erasers (ALKBH5 and FTO) reveals that the global m^6^A dynamic may be mainly driven by the cooperated regulation of METTL3 and ALKBH5 (Fig. 5B). To understand the m^6^A dynamics between the adjacent stages, we conducted a pairwise comparison to identify differentially methylated m^6^A peaks and genes. GO analysis reveals that the differentially m^6^A modification genes of LD30-LD60 and LD60-LD120 are enriched in typical skeletal muscle structures like sarcomere, myofibril, I band and contractile fiber, whereas the differentially m^6^A modification genes of D120-D180 are enriched in NADH and ATP related energy metabolic progress (Fig. 5F). Similarly, a re-analysis of the WGBS data of the corresponding samples revealed developmental stage-dependent DNA 5mC dynamics (Additional file 1: Fig. S7), as previously reported.

### Identification of key candidate genes under coordinated regulation of 5mC/m^6^A modifications

**Fig. 6:**
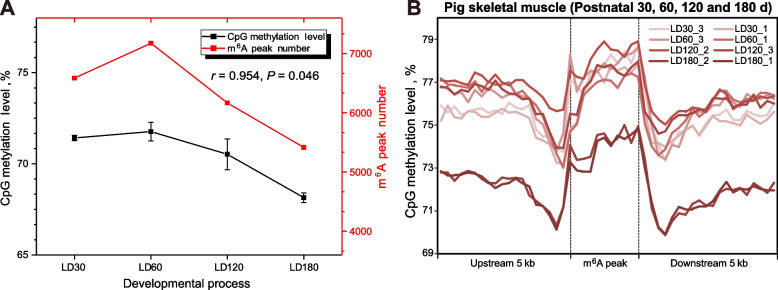
Genome/transcriptome-wide 5mC/m^6^A crosstalk effects in pig skeletal muscle of postnatal stages. **A** The average CpG methylation level shows a similar trend with the m^6^A peak number in the skeletal muscle along the postnatal developmental stages. **B** Enrichment pattern of 5mC modifications relative to m^6^A peaks. The m^6^A peaks, upstream 5 kb regions, downstream 5 kb regions were divided into 10, 20, 20 bins, respectively, to caculate its average methylation levels

By integrative analysis of the genome-wide 5mC and m^6^A sequencing data, we found a significant correlation between global DNA methylation level and m^6^A peak number during postnatal skeletal muscle development and growth (Fig. 6A, *r* = 0.954, *P*-value =0.046). In addition, we also observed significantly higher DNA methylation levels inside the m^6^A peaks than in the upstream and the downstream regions (Fig. 6B), which implicated an enrichment of DNA 5mC methylation in the corresponding RNA m^6^A modification sites in pig skeletal muscle. We then introduced a general linear model (GLM) to fit the 5mC/m^6^A interaction effects on mRNA expression for the Midnightblue module genes in the postnatal stages, under the hypothesis that the observed mRNA levels are affected by both 5mC and m^6^A modifications (see Materials and methods). We identified 15 regions in 9 genes with significant interaction items in the fitted model (Additional file 2: Table S2, 5mC/m^6^A interaction item *P*-value < 0.05). Intriguingly, five of these genes (*FBXO40*, *TMOD4*, *PGM1*, *CLIP1* and *ACTN3*) are involved in myogenesis and three of them (*PHKB*, *PFKFB1* and *ENO3*) are involved in the glycogen metabolic process (Additional file 2: Table S2).

## Discussion

Nucleotide methylation carries essential information for gene regulation, especially DNA 5-cytosine methylation (5mC) and RNA N6-adenosine methylation (m^6^A). Several previous studies indicated the essential roles of 5mC [23, 35] and m^6^A regulation [35, 36] during skeletal muscle development and growth, respectively. Our previous in-house study has revealed an overall decrease in DNA methylation level from the embryo to the adult pig skeletal muscle, which was highly correlated with the downregulated expression of DNMT1 [23]. We also described the m^6^A methylation landscape during prenatal skeletal muscle development and revealed essential roles in the regulation of myogenesis in another recent study [20]. This study focused on the crosstalk between these two critical epigenetic modifications in the postnatal development and growth of skeletal muscle. We validated the hypothesis that transcriptional (5mC) and posttranscriptional (m^6^A) regulation of hub genes is coordinated via protein-protein interaction of 5mC and m^6^A methylases, and disclosed a cooperative regulation of 5mC and m^6^A modifications on spatiotemporal gene expression in myogenesis.

The potential association of 5mC and m^6^A regulators was previously examined across 33 human cancer types, revealing their co-occurrence of genetic alterations and co-expression [21]. However, no data on 5mC and m^6^A modifications were available in that study. By taking advantage of a large-scale study on porcine skeletal muscle across 27 developmental stages, we performed a comprehensive evaluation of 5mC and m^6^A crosstalk in the present study. Initially, transcriptome data analysis indicated most 5mC and m^6^A regulators show a positively correlated expression in the skeletal muscle samples, leading to the establishment of 5mC/m^6^A EME by combining hub 5mC/m^6^A regulators. With this, we found the EME negatively correlated genes mainly function in myogenesis, hypoxia and glycolysis, which is in line with the physiological and structural changes of the muscle fiber in the postnatal growth stages. According to ATPase activity of the muscle fibers, the muscle fibers are categorized into four fiber types: MyHC I (slow-oxidative), MyHC IIa (fast-oxidative), MyHC IIx (intermediary to MyHC IIa and MyHC IIb), and MyHC IIb (fast-glycolytic) [37]. In pigs, oxidative metabolism represents the principal energy source during fetal life. At birth, glycolytic metabolism dramatically increases during the first postnatal weeks [38]. After that, the diameter of muscle fibers increases, and the muscle fibers lengthen rapidly [38]. Our results indicate that a down-regulated EME, which reflects both the pre- and post-transcriptional epigenetic status, may correlate to the enhanced gene co-expression in the postnatal muscle ontogenesis.

Moreover, immunofluorescence colocalization and co-IP experiments confirmed the interaction between 5mC and m^6^A methylases (Fig. 2). We further established an m^6^A epitranscriptome atlas of skeletal muscle and depicted the dynamics during postnatal skeletal muscle development for the first time in pigs. More m^6^A-contained genes and more variable changes of m^6^A modification in m^6^A-contained genes were identified in postnatal stages than prenatal stages [20]. These results indicated the crucial role of m^6^A modifications in postnatal skeletal muscle development, which is in line with previous documents [36, 39]. The m^6^A peak number and the DNA methylation level of pig skeletal muscle are changed simultaneously across different development stages. By integrating the 5mC and m^6^A sequencing data, co-localization of genome-wide 5mC and m^6^A modification was observed in porcine skeletal muscle. We further identified nine genes from the EME negative correlated module, which showed significant 5mC/m^6^A interaction effects on their mRNA transcription during postnatal skeletal muscle development and are crucial in myogenesis or glycogen metabolic process in muscle. Among them, *ACTN3* (actinin alpha 3) is a structural component of the sarcomeric Z line and is related to sarcopenia and physical fitness inactive older women [40, 41]. *PHKB* (phosphorylase kinase regulatory subunit beta) is involved in glycogen metabolism and associated with glycogen storage disease IXb, a disease also known as phosphorylase kinase deficiency in liver and muscle [42].

To our best knowledge, this is the first study suggesting protein-protein interactions between 5mC and m^6^A methylases, either directly or indirectly. We speculated that proteins like the histone deacetylase HDAC2 and the E3 ubiquitin protein ligase UHRF1 might act as adaptors to guide the interactions, as previous studies indicated they both could interact with the 5mC and m^6^A writers separately (Additional file 1 Fig. S8) [43–45]. Based on current data, we reasoned that 5mC methylase and m^6^A methylase are likely to add 5mC and m^6^A modifications simultaneously, thus enabling the synergistic transcriptional and post-transcriptional regulation of gene expression. Considering m^6^A is an essential mechanism for mRNA metabolism and protein translation, our results suggest that simultaneous nuclear events of transcriptional and translational regulation are coordinated for skeletal muscle cells. Despite this, the 5mC/m^6^A crosstalk needs further validation in different tissues and cell types from other species. Especially, although we revealed a number of key myogenesis-related genes that may be important targets of this synergistic regulation, further investigations to clarify the spatiotemporal mechanism of regulation and its developmental effects need to be conducted.

## Conclusions

In summary, our results lay a foundation for epigenetic regulation in skeletal muscle development and pave a new road for research on either basic muscle biology or animal breeding and muscle-related diseases. Since the development of demethylating drugs is currently a research hot spot, especially for RNA m^6^A inhibitors, the genes we supposed under tightly epigenetic regulation can serve as candidate targets for the treatment of muscle-related diseases.

## Supplementary Information

**Additional file 1: Fig. S1.** The whole design of this study. **Fig. S2.** Relative interaction intensities between the 5mC and m^6^A writer protein pairs based on the western blot images from co-IP assays. **Fig. S3.** Identification of WGCNA co-expression modules based on the top 25% expressing variant genes from the 27 prenatal and postnatal skeletal muscle development stages. **Fig. S4.** Potential regulation role of m^6^A/5mC EME on prenatal myogenesis of pigs. (A) EME positive correlated Lightyellow module and its gene expression heatmap. (B) GO process enrichment analysis of Midnightblue module genes. **Fig. S5.** Protein-Protein Interaction network among the Midnightblue module genes. **Fig. S6.** Distribution of m^6^A peaks across the whole transcript. **Fig. S7.** Dynamics of the DNA methylation during postnatal skeletal muscle development in pigs. (A) mRNA expression profiles of 5mC writers (DNMT1/DNMT3A/DNMT3B) and erasers (TET1/TET2/TET3). (B) Numbers of 5mC hypermethylated or hypomethylated genes from inter-stage comparisons (C) Top 5 GO terms enriched by the inter-stage differentially 5mC modification genes. **Fig. S8.** Possible indirect interactions between 5mC writers and m^6^A writers. (A) Protein-protein interaction network shows the proteins interacted with 5mC writers (DNMT1/3A/3B) in human beings. (B) Experiment evidence found in the published documents indicated the linker protein (UHRF1 and HDAC1) could interact with the m^6^A MTC proteins.

**Additional file 2: Table S1.** Summary of MeRIP-seq data in this study. **Table S2.** Genes expressed under significant 5mC and m^6^A interaction effects in postnatal skeletal muscle development. **Table S3–S6.** RNA expression data were used in this study. **Table S7–S10.** Consensus m^6^A peak identification of the four postnatal stages by exomePeak.

## Data Availability

All the sequencing data of MeRIP-seq have been deposited to Gene Expression Omnibus (GEO) at the National Center for Biotechnology Information (NCBI) under accession number GSE141943.

## Abbreviations

m^6^A: N6-methyladenosine
5mC: 5-methylcytosine
Co-IP: Co-immunoprecipitation
EME: Epigenetic module eigengenes
LD: *Longissimus dorsi*
GLM: General linear model
GO: Gene ontology
MeRIP-seq: Methylated RNA immunoprecipitation sequencing
RRBS: Reduced representation bisulfite sequencing
WGBS: Whole-genome bisulfite sequencing
WGCNA: Weighted correlation network analysis
