# Supplementary material for: Coordinated transcriptional and post-transcriptional epigenetic regulation during skeletal muscle development and growth in pigs
Source: J Anim Sci Biotechnol. 2022 Dec 1;13:146. doi: 10.1186/s40104-022-00791-3 (PMC9714148; doi:10.1186/s40104-022-00791-3)
Supplement: Supplementary file 1 — Additional file 1: Fig. S1. The whole design of this study. Fig. S2. Relative interaction intensities between the 5mC and m6A writer protein pairs based on the western blot images from co-IP assays. Fig. S3. Identification of WGCNA co-expression modules based on the top 25% expressing variant genes from the 27 prenatal and postnatal skeletal muscle development stages. Fig. S4. Potential regulation role of m6A/5mC EME on prenatal myogenesis of pigs. (A) EME positive correlated Lightyellow module and its gene expression heatmap. (B) GO process enrichment analysis of Midnightblue module genes. Fig. S5. Protein-Protein Interaction network among the Midnightblue module genes. Fig. S6. Distribution of m6A peaks across the whole transcript. Fig. S7. Dynamics of the DNA methylation during postnatal skeletal muscle development in pigs. (A) mRNA expression profiles of 5mC writers (DNMT1/DNMT3A/DNMT3B) and erasers (TET1/TET2/TET3). (B) Numbers of 5mC hypermethylated or hypomethylated genes from inter-stage comparisons (C) Top 5 GO terms enriched by the inter-stage differentially 5mC modification genes. Fig. S8. Possible indirect interactions between 5mC writers and m6A writers. (A) Protein-protein interaction network shows the proteins interacted with 5mC writers (DNMT1/3A/3B) in human beings. (B) Experiment evidence found in the published documents indicated the linker protein (UHRF1 and HDAC1) could interact with the m6A MTC proteins. [file 40104_2022_791_MOESM1_ESM.docx]

Supplementary material


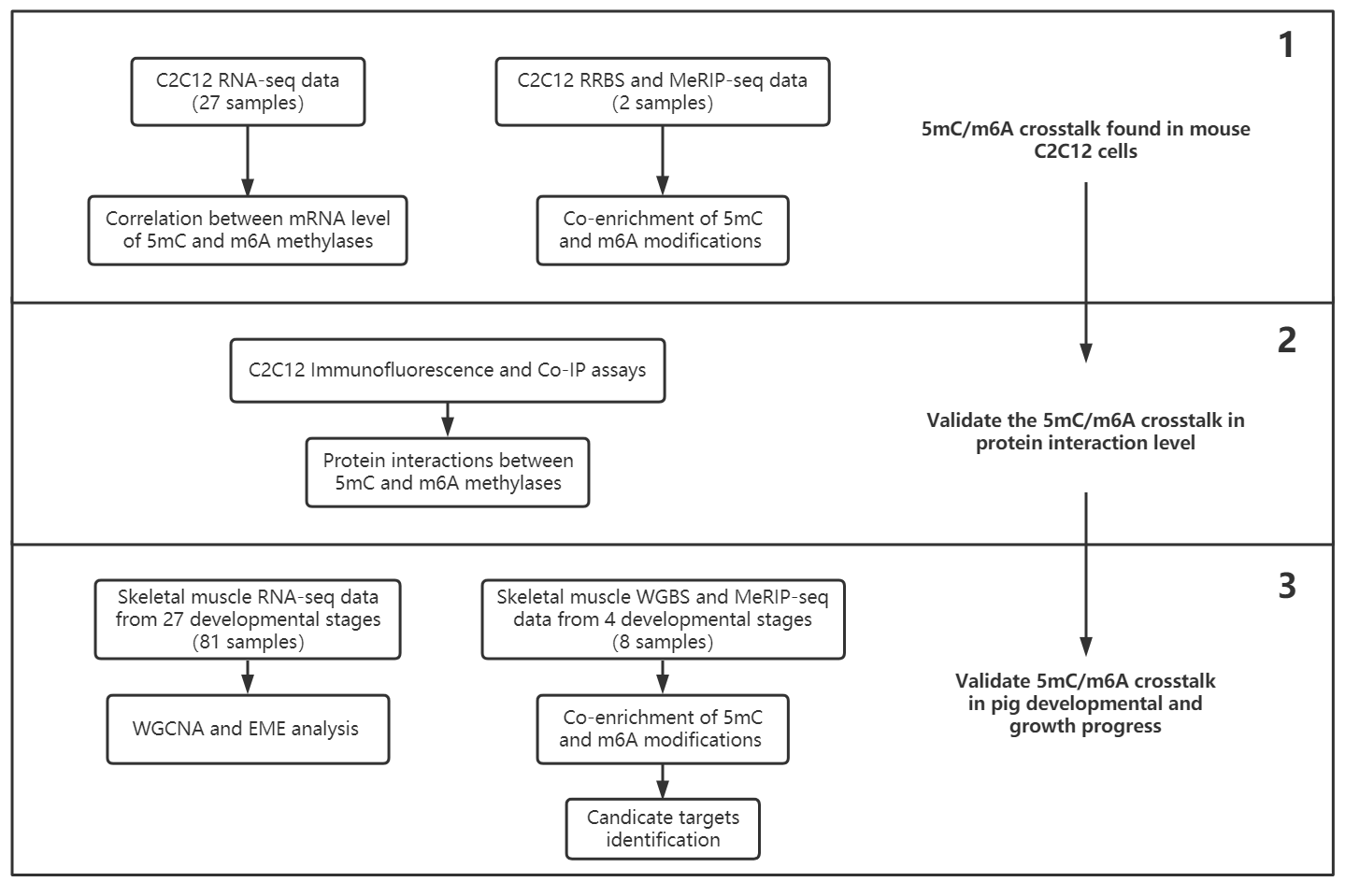


**Fig. S1** The whole design of this study


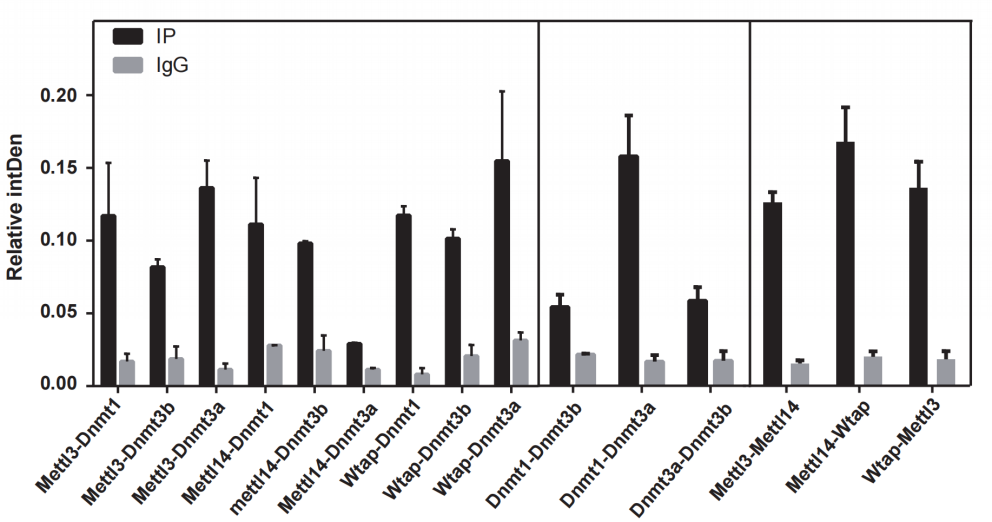


**Fig. S2** Relative interaction intensities between the 5mC and m6A writer protein pairs based on the western blot images from co-IP assays


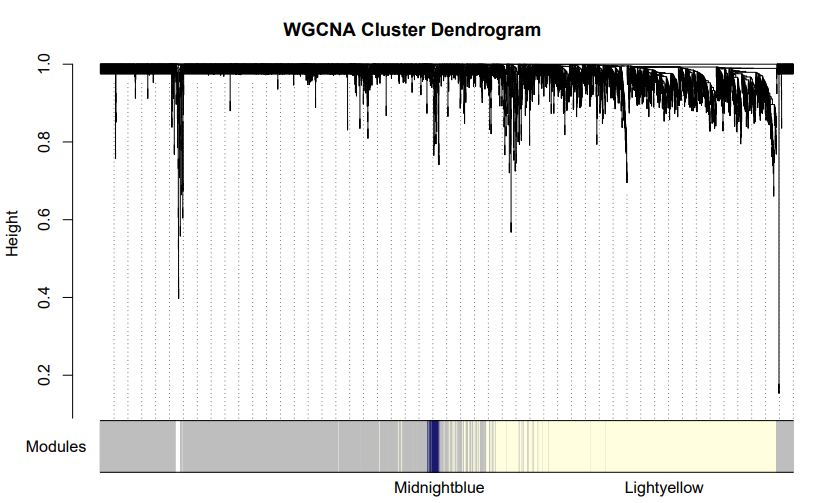


**Fig. S3** Identification of WGCNA co-expression modules based on the top 25% expressing variant genes from the 27 prenatal and postnatal skeletal muscle development stages


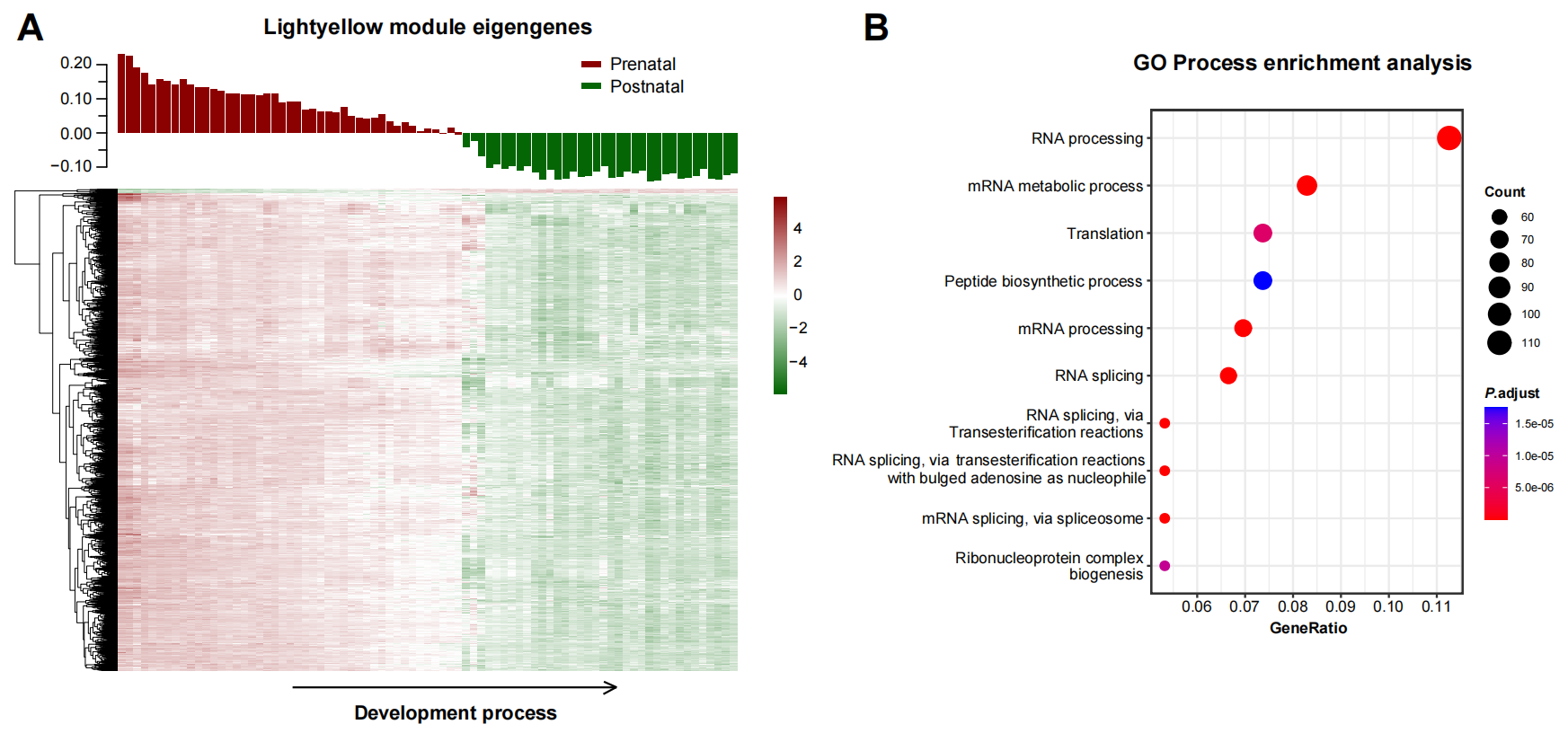


**Fig. S4** Potential regulation role of 5mC/m^6^A EMEs on prenatal myogenesis of pigs. (**A**) EME positive correlated Lightyellow module and its gene expression heatmap. (**B**) GO process enrichment analysis of Midnightblue module genes


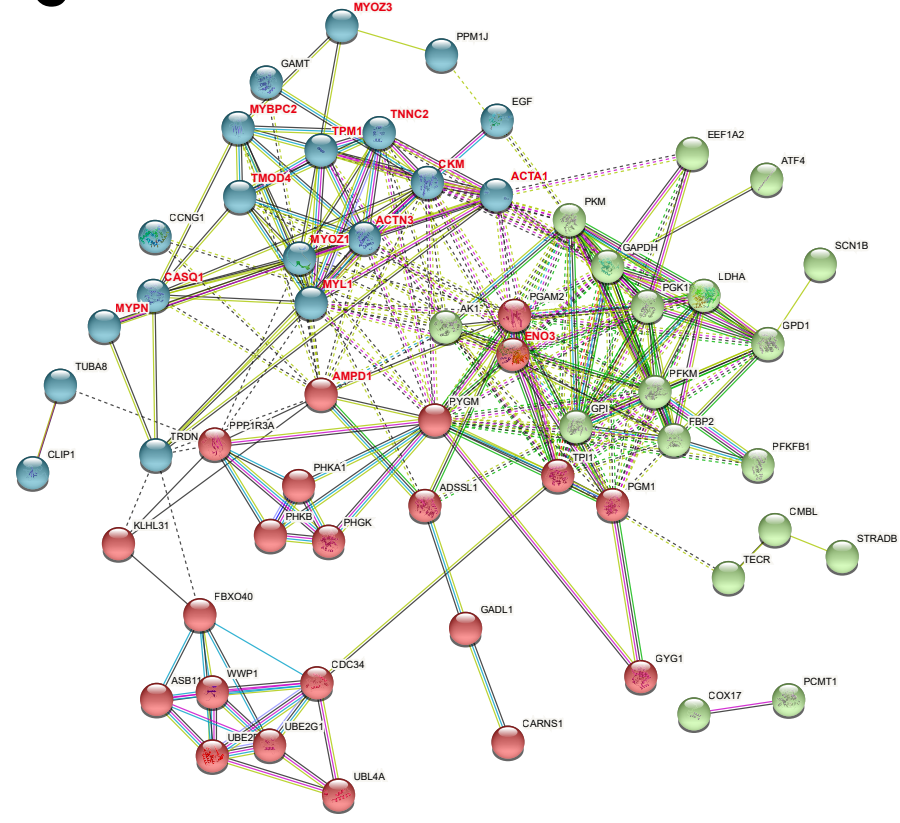


**Fig. S5** Protein-Protein Interaction network among the Midnightblue module genes


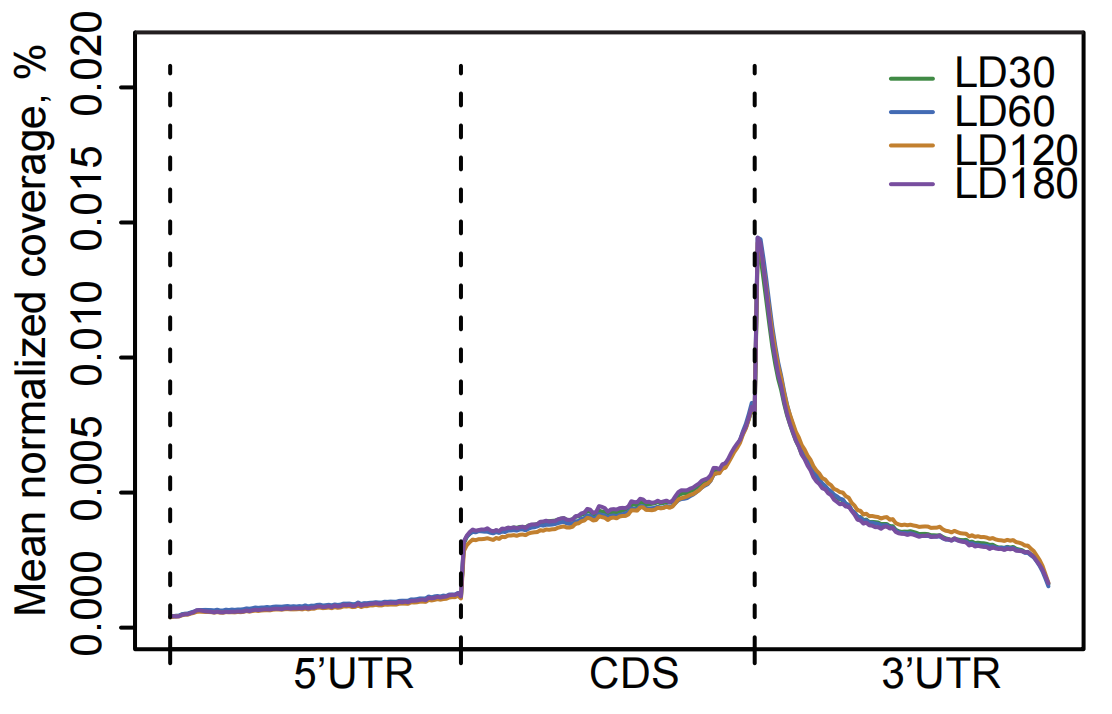


**Fig. S6** Distribution of m^6^A peaks across the whole transcript


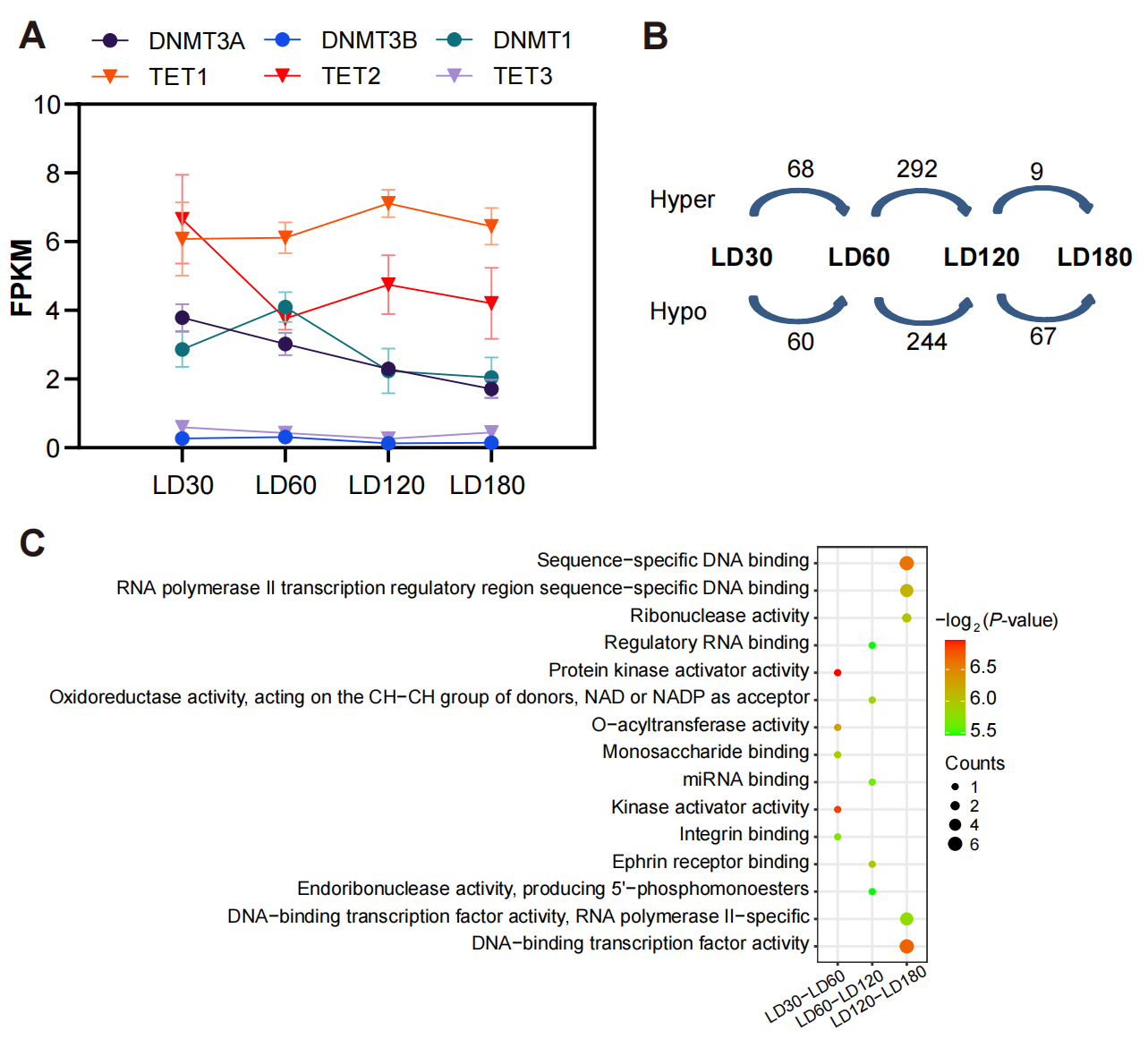


**Fig. S7** Dynamics of the DNA methylation during postnatal skeletal muscle development. (**A**) mRNA expression profiles of 5mC writers (DNMT1/DNMT3A/DNMT3B) and erasers (TET1/TET2/TET3). (**B**) Numbers of 5mC hypermethylated or hypomethylated genes from inter-stage comparisons (**C**) Top 5 GO terms enriched by the inter-stage differentially 5mC modification genes


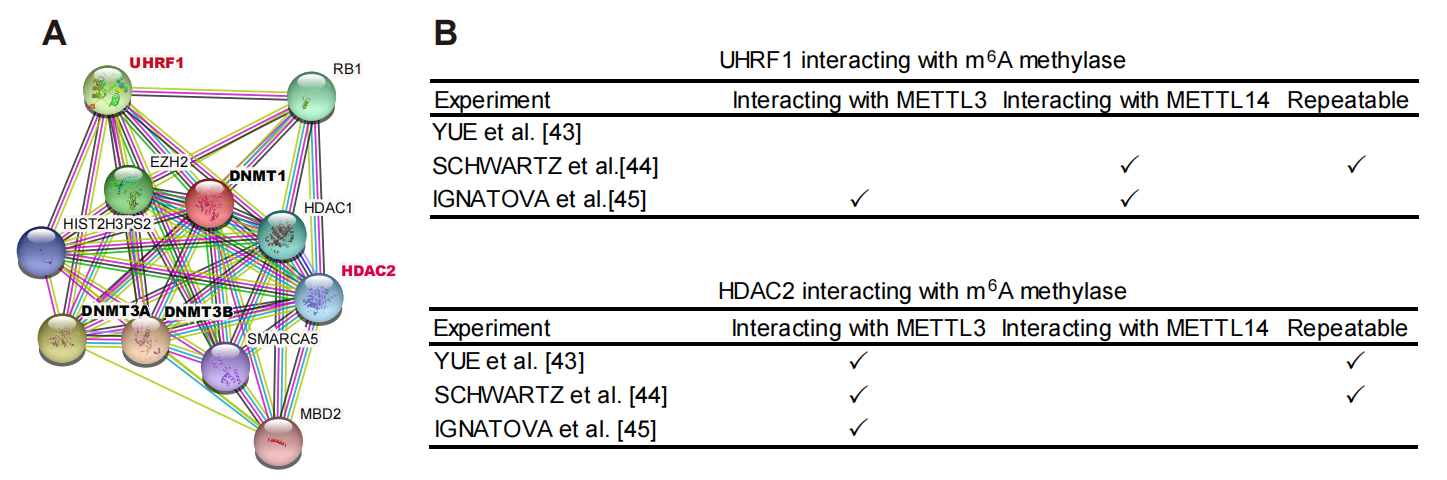


**Fig. S8** Possible indirect interactions between 5mC writers and m^6^A writers. (**A**) Protein-protein interaction network shows the proteins interacted with 5mC writers (DNMT1/3A/3B) in human beings. (**B**) Experiment evidence found in the published documents indicated the linker protein (UHRF1 and HDAC1) could interact with the m^6^A methyltransferase complex (MTC) proteins
